# Fasting Levels of High-Sensitivity Growth Hormone Predict Cardiovascular Morbidity and Mortality: The Malmö Diet and Cancer Study

**DOI:** 10.1016/j.jacc.2014.03.063

**Published:** 2014-10-07

**Authors:** Erik Hallengren, Peter Almgren, Gunnar Engström, Bo Hedblad, Margaretha Persson, Jennifer Suhr, Andreas Bergmann, Olle Melander

**Affiliations:** ∗ Department of Clinical Sciences, Lund University, Malmö, Sweden; † Department of Internal Medicine, Skåne University Hospital, Malmö, Sweden; ‡ ICI Immunochemical Intelligence GmbH, Berlin, Germany; § SphingoTec GmbH, Hohen Neuendorf, Germany; ‖ Waltraut Bergmann Foundation, Hohen Neuendorf, Germany

**Keywords:** cardiovascular disease, epidemiology, growth hormone, mortality, CAD, coronary artery disease, CHF, congestive heart failure, GH, growth hormone, GHD, growth hormone deficiency, HDL-C, high-density lipoprotein cholesterol, HR, hazard ratio, hs-GH, high-sensitivity growth hormone, LDL-C, low-density lipoprotein cholesterol, NRI, net reclassification improvement, NT-proBNP, N-terminal pro-brain natriuretic peptide

## Abstract

**Background:**

Both pathological excess and deficiency of growth hormone (GH) are associated with cardiovascular mortality.

**Objectives:**

The goal of this study was to test whether fasting levels of growth hormone measured with a high-sensitivity assay (hs-GH) predict cardiovascular morbidity and mortality at the population level.

**Methods:**

We studied 4,323 participants (age 46 to 68 years; mean age 58 years; 59% women) of the Swedish, population-based Malmö Diet and Cancer study examined in 1991 to 1994. Using multivariate-adjusted Cox proportional hazards models, we related baseline levels of fasting hs-GH to incidence of coronary artery disease, stroke, congestive heart failure, all-cause mortality, and cardiovascular mortality.

**Results:**

During a median follow-up of 16.2 years, hs-GH (hazard ratio [HR]/SD increment of natural logarithm of fasting hs-GH) was independently associated with increased risk of coronary artery disease (397 events; HR: 1.11; 95% confidence interval [CI]: 1.01 to 1.23; p = 0.04), stroke (251 events; HR: 1.18; 95% CI: 1.04 to 1.34; p = 0.01), congestive heart failure (107 events; HR: 1.25; 95% CI: 1.03 to 1.52; p = 0.02), all-cause mortality (645 events; HR: 1.17; 95% CI: 1.08 to 1.26; p < 0.001) and cardiovascular mortality (186 events; HR: 1.43; 95% CI: 1.24 to 1.66; p < 0.001). The addition of hs-GH to a model with conventional cardiovascular risk factors significantly reclassified risk, with a category-free net reclassification improvement (>0) of 0.542 (95% CI: 0.205 to 0.840) in cardiovascular mortality.

**Conclusions:**

Higher values of hs-GH were associated with an increased risk of cardiovascular morbidity and mortality.

Growth hormone (GH) is secreted from the anterior pituitary gland and exerts metabolic effects throughout the entire life, many (but not all) of which are mediated through insulin-like growth factor 1 (IGF-1) (1). GH and its role in the adult cardiovascular system have attracted growing interest during the last decades (1).

It is recognized that adults with hypopituitarism have increased mortality in cardiovascular and cerebrovascular diseases, which many 2, 3, 4, but not all (5), have attributed to growth hormone deficiency (GHD). Adult GHD patients have an adverse cardiovascular risk profile, with elevated low-density lipoprotein cholesterol (LDL-C) (6), body mass index (BMI) (6), and waist-hip ratio 6, 7, and decreased high-density lipoprotein cholesterol (HDL-C) (7). In these patients, GH substitution therapy has favorably affected some aspects of the cardiovascular risk profile 6, 8, 9, 10. Although the effects on glucose homeostasis may be negative 1, 8, 10, it can be tentatively assumed that GH substitution is beneficial. However, to date, there have been no large, randomized, placebo-controlled trials evaluating the effect of GH substitution on cardiovascular morbidity or mortality.

At the opposite end of the spectrum, patients with acromegaly, who suffer from excess secretion of growth hormone, also exhibit elevated mortality rates in cardiovascular and cerebrovascular diseases (11). Contributing factors to this mortality are higher prevalence of hypertension, diabetes, arrhythmias, and cardiomyopathy with hypertrophy of the heart 12, 13. The 1999 study by Takala et al. (14) further demonstrated the detrimental effects of GH excess (14). In this study, administration of high-dose GH to critically ill patients in an intensive care unit doubled mortality rates compared with placebo.

Most published reports concern patients with either GH excess or deficiency. However, the prospective study of French policemen, which began in the late 1960s and early 1970s with an 18-year follow-up, showed that higher fasting GH levels are associated with elevated risks for premature all-cause mortality and for cardiovascular mortality (15).

Accurate interpretation of plasma GH concentration has long been hampered by the release of GH in pulses over the majority of the day. However, in the morning hours, plasma GH concentration is relatively stable 16, 17, 18 but is low and sometimes undetectable with conventional GH assays. We used a new high-sensitivity assay for plasma growth hormone measurements (hs-GH), which is able to quantify with high precision even the normally low range of GH levels (19). Given the limited knowledge on the relationship between fasting plasma GH concentration and development of cardiovascular morbidity and mortality, we measured hs-GH in fasted plasma in a large, healthy population and related hs-GH to risk of coronary artery disease (CAD), stroke, congestive heart failure (CHF), all-cause mortality, and cardiovascular mortality during long-term follow-up.

## Methods

The MDC (Malmö Diet and Cancer) study is a population-based, prospective cohort of 28,449 individuals examined between 1991 and 1996 (20). From this cohort, a random sample, examined between November 1991 and February 1994 (n = 6,103), was included in the MDC cardiovascular cohort, with the primary aim of studying the epidemiology of carotid artery disease (21). After exclusion of individuals lacking values from fasting plasma samples and thus missing data on prevalence of diabetes mellitus or fasting values of HDL-C, LDL-C, or GH, the cohort consisted of 4,452 persons. Of these, 129 individuals had a history of CAD, stroke, or CHF and were excluded from further analysis. Thus, 4,323 persons, age 46 to 68 years, remained to comprise the primary study cohort.

All participants provided written consent, and the Ethical Committee at Lund University, Lund, Sweden approved the study.

At baseline (1991 to 1996), participants underwent a medical history, physical examination, and laboratory assessment. Diabetes mellitus was defined as either self-report of a physician diagnosis, use of diabetes medication, or fasting venous whole blood glucose >6.0 mmol/l (109 mg/dl). Levels of HDL-C, total cholesterol, creatinine, glycated hemoglobin (HbA1c), and insulin were measured according to standard procedures at the Department of Clinical Chemistry, University Hospital of Malmö. LDL-C levels were calculated according to the Friedewald formula. Levels of N-terminal pro-brain natriuretic peptide (NT-proBNP) were determined using the Dimension RxL automated NT-proBNP method (Siemens Diagnostics, Nürnberg, Germany) (22).

All samples of plasma and whole blood were obtained after overnight fasting, and samples were drawn between 7:30 am and 9:00 am. Further details about the clinical examination can be found in the Online Appendix.

GH levels were measured in stored fasting plasma samples, which were frozen immediately to −80°C at the Malmö Diet and Cancer Study–Cardiovascular Cohort baseline examination. The measurement was made with a high-sensitivity chemiluminescence sandwich immunoassay similar to 1 previously described (SphingoTec GmbH, Borgsdorf, Germany) (19). The analytical assay sensitivity (mean relative light units of 20 determinations of GH free sample + 2 SD) was 2 pg/ml GH. The functional assay sensitivity (<20% interassay coefficient of variation) was 10 pg/ml. The assay is further described in the Online Appendix. Individuals with a GH value equal to 0 (n = 13) were censored in all analyses and are not included in the count of 4,323 persons discussed earlier.

We examined 5 primary outcomes: CAD, stroke, CHF, cardiovascular mortality, and total mortality. The endpoints were retrieved through record linkage of the personal identification number of each Swedish individual and the Swedish Hospital Discharge Register, the Swedish Cause of Death Register, the Stroke in Malmö Register, and the Swedish Coronary Angiography and Angioplasty Registry. These registries were previously described and validated for classification of outcomes 23, 24, 25, 26. CAD was defined as fatal or nonfatal myocardial infarction, death due to ischemic heart disease, percutaneous coronary intervention, or coronary artery bypass grafting, whichever came first. Stroke was defined as fatal or nonfatal stroke. Congestive heart failure was defined as a fatal or nonfatal episode of CHF. Cardiovascular mortality was defined as a cardiovascular disease as the cause of death. Codes, which are the basis for the events, are specified in the Online Appendix. Follow-up for outcomes was extended to June 30, 2009.

Fasting values of hs-GH, insulin, and NT-proBNP exhibited a right-skewed distribution and were transformed into natural logarithms before analysis. To determine if GH displayed any correlation with traditional cardiovascular risk factors (27), cross-sectional analyses were performed at baseline using linear regression models with standardized logarithmic hs-GH as the dependent variable and age, sex, current smoking, antihypertensive medication, diabetes mellitus, and the standardized values for: systolic blood pressure, BMI, HDL-C, and LDL-C as independent variables entered either separately (crude) or simultaneously (multivariate adjusted).

Multivariable Cox proportional hazard models were performed to examine the association between GH and incidence of cardiovascular events and mortality. All models were adjusted for age, sex, systolic blood pressure, use of antihypertensive medication, current smoking, diabetes mellitus, BMI, and levels of LDL-C and HDL-C. We confirmed that the proportionality of hazards assumption was met using Schoenfeld residuals. Hazard ratios (HRs) for GH were expressed per 1-SD increment of the natural logarithm of hs-GH. Values of hs-GH were standardized separately in each sex before being used in sex-combined models, due to the large difference of hs-GH between the sexes. To establish if there were any significant sex differences, a sex interaction test was performed. A limited number of other cardiovascular risk factors or factors assumed to be associated with GH (NT-proBNP, creatinine, waist circumference, body fat percentage, insulin, and HbA1c) were then added to the models to evaluate the stability of the results.

To evaluate if GH had any predictive potential we calculated the category-free net reclassification improvement (NRI) (>0) according to previously described methods (28). We used multivariable risk scores with the parameters mentioned in the preceding text to estimate the risk of developing a cardiovascular event and then examined whether the addition of hs-GH improved the model and reclassified subjects, thus obtaining the NRI (>0). Up-classification in the risk of individuals who developed an event or down-classification of individuals who did not develop an event improves the risk model, whereas the opposite deteriorates the model. Calculation of C statistics (Harrell’s) (29) was used to assess model discrimination.

A post-hoc analysis was made examining the case fatality of myocardial infarctions versus hs-GH. Case fatality was defined as death within 24 h of the event. Odds ratios were calculated by means of logistic regression analyses and adjusted for the risk factors included in our original model, with the addition of year of event and age at event replacing age at baseline. Odds ratios for GH were expressed per 1-SD increment of the natural logarithm of hs-GH.

All analyses were made with Stata software version 11 (StataCorp, College Station, Texas). A 2-sided p value <0.05 was considered statistically significant.

## Results

Women had significantly higher fasting values of hs-GH than men (Table 1). Individuals excluded from the study due to missing plasma samples or prevalent cardiovascular disease differed slightly in their baseline characteristics (Online Table 1).

**Table 1:** Clinical Characteristics of the Study Population

	Male	Female
n (% of whole cohort)	1,778 (41.1)	2,545 (58.9)
Age, yrs	57.8 ± 6.0	57.5 ± 5.9
Systolic blood pressure, mm Hg	144 ± 19	140 ± 19
Body mass index, kg/m^2^	26.1 ± 3.4	25.5 ± 4.2
Antihypertensive therapy	279 (15.7)	384 (15.1)
Diabetes mellitus	197 (11.1)	162 (6.4)
LDL-C, mmol/l	4.11 ± 0.89	4.20 ± 1.05
HDL-C, mmol/l	1.22 ± 0.30	1.51 ± 0.37
Current smokers	491 (27.6)	650 (25.5)
Growth hormone, μg/l	0.11 (0.06–0.33)	1.22 (0.40–3.15)∗

Values are n (%), mean ± SD, or median (interquartile range).

HDL-C = high-density lipoprotein cholesterol; LDL-C = low-density lipoprotein cholesterol.

∗ Significant difference vs. males (p < 0.001). The p value for differences in other variables not calculated.

With the exception of age in women and systolic blood pressure and antihypertensive medication in men (Online Table 2), most variables were significant determinants of hs-GH in the crude regression models. In the adjusted models, age, current smoking, and HDL-C had a significant positive correlation with hs-GH, whereas LDL-C and BMI exhibited significant negative correlation in both sexes (Table 2, Online Tables 3 and 4). Prevalence of diabetes mellitus had a positive correlation with hs-GH in males and a negative correlation in females. The multiple regression models had an R^2^ value of 9.5% for males and 11.8% for females.

**Table 2:** Results From Multiple Linear Regression Models Examining Correlations Between Fasting Values of Growth Hormone and Traditional Cardiovascular Risk Factors

Risk factor	Male			Female		
	β Coefficient∗	95% CI	p Value	β Coefficient∗	95% CI	p Value
Age	0.02	0.01 to 0.03	<0.001	0.02	0.01 to 0.02	<0.001
Systolic blood pressure	0.02	−0.03 to 0.07	0.38	−0.04	−0.08 to 0.00	0.04
Antihypertensive medication	0.13	0.00 to 0.26	0.05	0.07	−0.03 to 0.18	0.18
BMI	−0.07	−0.12 to −0.03	0.003	−0.25	−0.29 to −0.21	<0.001
Current smoking	0.29	0.19 to 0.39	<0.001	0.19	0.10 to 0.27	<0.001
LDL-C	−0.09	−0.13 to −0.04	<0.001	−0.10	−0.14 to −0.06	<0.001
HDL-C	0.20	0.15 to 0.25	<0.001	0.08	0.04 to 0.12	<0.001
Diabetes mellitus	0.21	0.07 to 0.36	0.004	−0.16	−0.31 to 0.00	0.05

BMI = body mass index; other abbreviations as in Table 1.

∗ The β coefficients are expressed as the increment of standardized values of the natural logarithm of high-sensitivity growth hormone per 1 increment of standardized values (or presence of dichotomized risk factor) of the risk factor in question. NB age is not standardized. BMI (weight [kg] divided by height [m^2^]), systolic blood pressure, and fasting values of HDL-C and LDL-C are standardized. Prevalence of diabetes mellitus, current smoking, and use of antihypertensive medication are dichotomous variables.

Median follow-up time ranged from 16.1 years (interquartile range: 15.4 to 16.7 years) in CAD to 16.2 years (interquartile range: 15.6 to 16.7 years) in the mortality analyses. Total follow-up time was more than 66,000 person-years. All of the Cox proportional hazards models were significant when analyzing the whole cohort, with increasing hs-GH levels associated with elevated cardiovascular morbidity and mortality, independent of other cardiovascular risk factors (Table 3). HRs for each SD increase of baseline hs-GH ranged from 1.11 in CAD to 1.43 in cardiovascular mortality. A total of 4 of 5 male analyses were significant, with HRs ranging from 1.17 in CAD to 1.44 in cardiovascular mortality, and the 5th analysis, which was CHF, was nonsignificant. In females, HRs were significant for cardiovascular mortality (1.45) and CHF (1.48). A significant sex interaction was found in the analysis of total mortality (p = 0.02).

**Table 3:** Multivariate-Adjusted Cox Proportional Hazards Models for Baseline Fasting Value of hs-GH Versus Incidence of CAD, Stroke, CHF, All-Cause Mortality, and Cardiovascular Mortality

Event	n	Sex	Continuous∗			Q1†	Q2†			Q3†			Q4†			Trend†		
			HR	95% CI	p Value	HR‡	HR∗	95% CI	p Value	HR‡	95% CI	p Value	HR‡	95% CI	p Value	HR‡	95% CI	p Value
CAD	397	All	1.11	1.01–1.23	0.04	1.00 (ref)	1.10	0.83–1.47	0.50	1.05	0.77–1.42	0.76	1.33	0.99–1.79	0.05	1.09	0.99–1.19	0.08
	247	Male	1.17	1.04–1.33	0.01	1.00 (ref)	0.92	0.64–1.33	0.66	0.93	0.63–1.39	0.74	1.45	1.01–2.08	0.05	1.14	1.01–1.29	0.03
	150	Female	1.02	0.86–1.21	0.81	1.00 (ref)	1.41	0.90–2.21	0.13	1.23	0.76–1.97	0.39	1.06	0.63–1.78	0.83	1.00	0.86–1.17	0.96
Stroke	251	All	1.18	1.04–1.34	0.01	1.00 (ref)	1.16	0.80–1.70	0.43	1.37	0.94–2.01	0.10	1.52	1.04–2.23	0.03	1.15	1.02–1.30	0.02
	132	Male	1.21	1.02–1.43	0.02	1.00 (ref)	1.03	0.61–1.74	0.92	1.03	0.59–1.81	0.91	1.53	0.91–2.56	0.11	1.16	0.98–1.36	0.08
	119	Female	1.16	0.95–1.41	0.15	1.00 (ref)	1.32	0.77–2.27	0.32	1.78	1.05–3.02	0.03	1.51	0.85–2.71	0.16	1.17	0.98–1.39	0.08
CHF	107	All	1.25	1.03–1.52	0.02	1.00 (ref)	1.42	0.76–2.67	0.27	2.19	1.20–4.01	0.01	1.90	1.01–3.56	0.05	1.24	1.03–1.49	0.02
	56	Male	1.07	0.83–1.39	0.59	1.00 (ref)	1.08	0.44–2.65	0.87	1.91	0.82–4.48	0.14	1.42	0.60–3.36	0.43	1.15	0.90–1.48	0.27
	51	Female	1.48	1.08–2.04	0.02	1.00 (ref)	1.77	0.74–4.25	0.20	2.45	1.04–5.78	0.04	2.28	0.89–5.82	0.08	1.30	0.99–1.71	0.05
Total mortality	645	All	1.17	1.08–1.26	<0.001	1.00 (ref)	0.93	0.73–1.19	0.58	1.27	1.00–1.61	0.05	1.45	1.15–1.83	0.002	1.16	1.08–1.25	<0.001
	341	Male	1.25	1.13–1.39	<0.001	1.00 (ref)	1.19	0.82–1.72	0.36	1.66	1.15–2.40	0.006	2.05	1.44–2.92	<0.001	1.29	1.16–1.43	<0.001
	304	Female	1.04	0.92–1.18	0.50	1.00 (ref)	0.77	0.55–1.08	0.13	1.05	0.76–1.44	0.77	1.02	0.73–1.42	0.93	1.04	0.93–1.16	0.52
CVD mortality	186	All	1.43	1.24–1.66	<0.001	1.00 (ref)	1.33	0.79–2.24	0.28	2.13	1.30–3.51	0.003	2.83	1.74–4.61	<0.001	1.43	1.24–1.65	<0.001
	105	Male	1.44	1.20–1.72	<0.001	1.00 (ref)	1.51	0.71–3.20	0.28	2.26	1.08–4.73	0.03	3.32	1.65–6.70	<0.001	1.49	1.23–1.81	<0.001
	81	Female	1.45	1.12–1.88	0.004	1.00 (ref)	1.13	0.54–2.36	0.75	2.04	1.04–4.03	0.04	2.36	1.16–4.80	0.02	1.38	1.11–1.72	0.004

1 model with continuous values of high-sensitivity growth hormone (hs-GH) and 1 with cohort split into sex-specific quartiles of hs-GH. In total, 4,323 (1,778 males, 2,545 females) individuals available for analysis in all models.

CAD = coronary artery disease; CHF = congestive heart failure; CVD = cardiovascular disease; other abbreviations as in Table 1.

∗ Hazard ratios (HRs) in continuous (95% confidence interval [CI]) are expressed per 1 SD increment of the natural logarithm of hs-GH. Values of hs-GH are standardized separately in men and women, and this same sex-specific standardization is used in the combined analyses.

† Quartile 1 (Q1) represents the quartile with the lowest values of fasting hs-GH. Males and females are divided into the quartiles separately, which makes the male/female ratio similar in the quartiles, but the cut-off values different in the different sexes.

‡ HRs (95% CI) in quartile analysis are expressed vs. the HR in Q1, except in “trend” where HR is expressed per 1 increment of quartile. Variables adjusted for in the analysis: age, systolic blood pressure, use of antihypertensive medication, BMI (weight [kg] divided by height [m^2^]), prevalence of diabetes mellitus, current smoking, and fasting values of HDL-C and LDL-C. In addition, adjusted for sex in the sex-combined analyses.

When comparing the quartile with the highest values of hs-GH with the bottom quartile, HRs ranged from 1.33 (95% confidence interval [CI]: 0.99 to 1.79; p = 0.05) in CAD to 2.83 (95% CI: 1.74 to 4.61; p < 0.001) in cardiovascular mortality (Table 3). Male HRs were significant in CAD (HR: 1.45; 95% CI: 1.01 to 2.08; p = 0.05), total mortality (HR: 2.05; 95% CI: 1.44 to 2.92; p < 0.001), and cardiovascular mortality (HR: 3.32; 95% CI: 1.65 to 6.70; p < 0.001). Female quartile analyses were significant in cardiovascular mortality (HR: 2.36; 95% CI: 1.16 to 4.80; p = 0.02) when comparing the highest quartile of hs-GH to the lowest.

We investigated whether our findings were independent of known GH-associated variables and other cardiovascular risk factors by adding insulin, HbA1c, creatinine, NT-proBNP, waist circumference, and body fat percentage to the different Cox models mentioned previously. Generally, these variables had only minor effects on HRs or CIs, with NT-proBNP and HbA1c having the largest effects, resulting in a slight attenuation of some results (Online Tables 5 and 6). If the analyses were performed crude, the results were similar, but not identical (Online Table 7, Online Figures 1 to 9).

In the reclassification analyses, the NRI (>0) was significant for the whole cohort in stroke (0.179; 95% CI: 0.003 to 0.293), total mortality (0.207; 95% CI: 0.020 to 0.383), and cardiovascular mortality (0.542; 95% CI: 0.205 to 0.840) (Table 4, Online Table 8). The 2 mortality analyses were significant for males, whereas the female analyses were significant in cardiovascular mortality. Improvement mostly stemmed from down-classification of nonevents in the total mortality analysis and up-classification of events in cardiovascular mortality. To evaluate the reclassification model, we tried adding creatinine and NT-proBNP to the basic model before calculating the NRI (>0) for hs-GH. This attenuated the NRI (>0) in stroke and total mortality, whereas the association in cardiovascular mortality remained the same (Table 4).

**Table 4:** Reclassification of Risk Estimates (Category-Free NRI [>0]) for Addition of Growth Hormone to 1 Model With Conventional Cardiovascular Risk Factors (Basic) and 1 Model With 2 Additional Risk Factors∗

		Basic			Basic + NT-proBNP + Creatinine
		Overall NRI (95% CI)	Events (95% CI)∗	Nonevents (95% CI)∗	Overall NRI (95% CI)∗
CAD	All	0.081 (−0.035 to 0.185)	—	—	—
	Male	0.143 (−0.011 to 0.314)	—	—	—
	Female	0.144 (−0.145 to 0.313)	—	—	—
Stroke	All	0.179 (0.003 to 0.293)	0.082 (−0.053 to 0.162)	0.097 (−0.451 to 0.156)	0.155 (−0.032 to 0.288)
	Male	0.178 (−0.015 to 0.354)	—	—	—
	Female	0.191 (−0.047 to 0.383)	—	—	—
CHF	All	0.115 (−0.122 to 0.376)	—	—	—
	Male	0.142 (−0.148 to 0.407)	—	—	—
	Female	0.248 (−0.125 to 0.530)	—	—	—
Total mortality	All	0.207 (0.020 to 0.383)	0.084 (−0.060 to 0.239)	0.122 (0.058 to 0.178)	0.208 (−0.008 to 0.449)
	Male	0.539 (0.131 to 0.983)	0.116 (−0.131 to 0.482)	0.422 (0.258 to 0.655)	0.538 (0.129 to 1.099)
	Female	0.041 (−0.057 to 0.179)	—	—	—
Cardiovascular mortality	All	0.542 (0.205 to 0.840)	0.394 (0.089 to 0.727)	0.147 (0.068 to 0.288)	0.564 (0.180 to 0.864)
	Male	0.853 (0.166 to 1.633)	0.411 (−0.083 to 1.064)	0.442 (0.203 to 0.711)	0.872 (0.177 to 1.571)
	Female	0.332 (0.137 to 0.564)	0.277 (0.110 to 0.466)	0.055 (−0.029 to 0.145)	0.370 (0.032 to 0.625)

Variables included in the original model: sex, age, systolic blood pressure, body mass index, antihypertensive medication, current smoking, diabetes mellitus, LDL-C, and HDL-C.

NRI = net reclassification improvement; NT-proBNP = N-terminal probrain natriuretic peptide; other abbreviations as in Table 1, Table 3.

∗ Data in “events,” “nonevents,” and the extended model are only shown in the outcomes where the basic overall NRI is significant.

Overall, the effects on the C-statistics when adding GH to the basic model were modest. In analysis of cardiovascular mortality the C-statistics increased from 0.783 (95% CI: 0.752 to 0.814) to 0.794 (95% CI: 0.763 to 0.825) in the whole cohort, 0.751 (95% CI: 0.705 to 0.797) to 0.764 (95% CI: 0.719 to 0.809) in males, and 0.797 (95% CI: 0.751 to 0.844) to 0.807 (95% CI: 0.760 to 0.854) in females (Table 5).

**Table 5:** Improvement of Discrimination (C-Statistics) of 10-Year Risk Estimates for Addition of Growth Hormone to a Model With Conventional Cardiovascular Risk Factors

Outcome	Sex	Basic (95% CI)	+GH (95% CI)
CAD	All	0.748 (0.726–0.771)	0.749 (0.726–0.772)
	Male	0.696 (0.665–0.726)	0.698 (0.667–0.729)
	Female	0.744 (0.707–0.782)	0.744 (0.707–0.782)
Stroke	All	0.753 (0.726–0.780)	0.757 (0.730–0.784)
	Male	0.745 (0.708–0.781)	0.749 (0.712–0.785)
	Female	0.749 (0.709–0.789)	0.752 (0.713–0.791)
CHF	All	0.782 (0.739–0.825)	0.786 (0.743–0.828)
	Male	0.784 (0.725–0.845)	0.784 (0.724–0.844)
	Female	0.789 (0.724–0.853)	0.801 (0.737–0.865)
Total mortality	All	0.711 (0.691–0.730)	0.714 (0.695–0.734)
	Male	0.700 (0.673–0.727)	0.709 (0.683–0.736)
	Female	0.703 (0.674–0.732)	0.703 (0.674–0.733)
Cardiovascular mortality	All	0.783 (0.752–0.814)	0.794 (0.763–0.825)
	Male	0.751 (0.705–0.797)	0.764 (0.719–0.809)
	Female	0.797 (0.751–0.844)	0.807 (0.760–0.854)

Harrell’s c-statistic. Area under the receiver-operating characteristics curve in basic model without hs-GH (basic) and the basic model with addition of hs-GH (+GH). Variables included in the original model: sex, age, systolic blood pressure, body mass index, antihypertensive medication, current smoking, diabetes mellitus, LDL-C, and HDL-C.

Abbreviations as in Table 1, Table 3.

A post-hoc analysis of the 24-h case fatality of myocardial infarctions versus hs-GH was performed to investigate the strong relationship between hs-GH and cardiovascular mortality. In a multivariate-adjusted logistic regression, the odds ratio per 1-SD increment of fasting hs-GH at baseline was 1.54 (310 events, 69 fatal; 95% CI: 1.13 to 2.09; p = 0.006) in the total cohort, 1.50 (187 events, 37 fatal; 95% CI: 1.00 to 2.23; p = 0.05) among males, and 1.67 (123 events, 32 fatal; 95% CI: 0.98 to 2.85; p = 0.06) among females.

## Discussion

We examined whether fasting values of hs-GH predict cardiovascular morbidity and mortality in a large population-based cohort free from CAD, CHF, and stroke at baseline with a longitudinal follow-up time exceeding 66,000 person-years. Increasing fasting hs-GH levels were associated with higher incidence of CAD, stroke, CHF, all-cause mortality, and cardiovascular mortality, independent of traditional cardiovascular risk factors.

Adult GHD patients have an adverse cardiovascular risk factor profile 6, 7 and were reported to have an increased risk of all-cause and cardiovascular mortality 2, 3, 4. Although GH substitution ameliorates some aspects of the cardiovascular risk factor profile 6, 8, 9, 10, there are no randomized placebo-controlled trials evaluating this therapy in relation to hard endpoints such as cardiovascular morbidity and mortality. Interestingly, at the population level, we found that patients with low hs-GH levels resembled GHD patients in having an unfavorable body composition and lipoprotein profile (Online Table 9). However, the relationship between hs-GH and cardiovascular morbidity and mortality was the opposite of that expected from the observations in GHD patients: subjects with low hs-GH had low risk, whereas subjects with high hs-GH had high risk. Although our study was performed in healthy subjects and does not prove a causal relationship between increasing GH and cardiovascular morbidity and mortality, it does cast doubts on whether GH replacement therapy in adult GHD patients is beneficial for cardiovascular health. Most commonly, GHD patients are deficient for a reason, and previous cranial irradiation, surgery, and other causes may affect mortality rates independently from GHD. Rather, our results, together with the only previous study of fasting GH in relation to all-cause and cardiovascular mortality in healthy subjects (15), suggest that elevated fasting GH is an independent risk factor for cardiovascular morbidity and mortality, and thus call for randomized placebo-controlled trials of GH replacement therapy to determine its effect on cardiovascular morbidity and mortality.

Today, GH use is not limited to individuals with GHD. For example, GH has been used as an antiaging therapy, and athletes also use it. A systematic review concluded that GH could not be recommended as antiaging therapy, and our results further highlight the impropriety and possible dangers of this practice (30). The use of GH in sports is classed as doping, and its performance-enhancing capabilities are, in many areas, doubtful (31). Physical exercise results in secretion of GH; however, regular exercise has not been shown to alter the baseline resting levels (32), which may indicate that regular physical activity does not affect the fasting value of GH.

As would be expected, studies in rodents showed that mice overexpressing GH genes have a drastically shortened life span (33). What is more surprising is that mice that are either missing the GH receptor (growth hormone receptor knockout, GHRKO) or Ames mice (deficient in GH, prolactin, and thyroid-stimulating hormone) live longer than normal mice 34, 35 while being obese compared with their siblings 36, 37. Although the relevance of these experimental data for humans is unclear, they suggest a link between reduced GH signaling and a phenotype of obesity and longer life span. This somewhat resembles the situation in humans with low hs-GH who have higher BMIs, waist circumference, and body fat, but live longer in comparison with subjects with high hs-GH (Central Illustration).

**Central Illustration:**
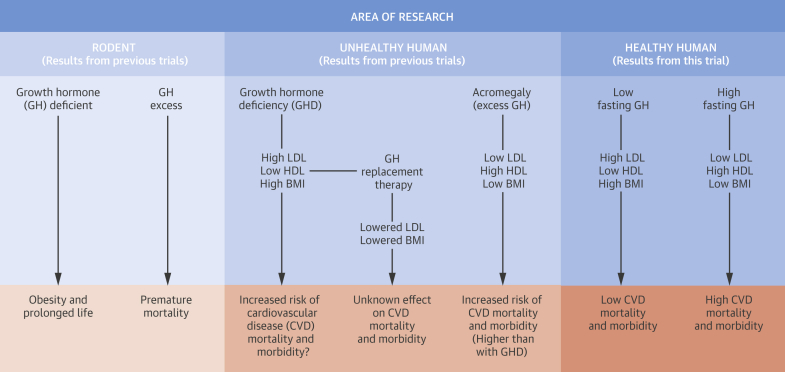
Brief Summary of the Relation Between GH, Risk Factors, and Cardiovascular Diseases Schematic figure shows links between GH levels and cardiovascular diseases found in research on animals and humans. BMI = body mass index; HDL = high-density lipoprotein; LDL = low-density lipoprotein.

The outcome with the strongest independent relationship to baseline fasting hs-GH was cardiovascular mortality. In this outcome, adding hs-GH to traditional cardiovascular risk factors resulted in increments of the NRI (>0) of 0.542 in the total cohort, suggesting that addition of hs-GH improved the basic predictive model. Even with the addition of creatinine and NT-proBNP, the association remained significant. Interestingly, the NRI (>0) in cardiovascular mortality was driven to a larger extent by up-classification of subjects with a cardiovascular death than by down-classification of healthy subjects. Even though the clinical use of hs-GH for prediction would be associated with many drawbacks, these results still indicate that the predictive possibility is not negligible.

In general, we found a stronger relationship between hs-GH in men than in women. There are several potential explanations for this. Our finding that women have in the order of 10-fold higher values of fasting GH than men has been previously described (38). The 24-h profiles of GH secretion in women were shown to be more irregular and to have shorter oscillatory periods and higher trough values than in men 39, 40, 41. It should be kept in mind that fasting GH values are not a standard clinical test, and it is not known how these values relate to overall GH production. When examining 24-h GH profiles from previous publications, morning values in men appear quite low and stable, whereas the variation in women is larger 16, 17, 18. It can thus be speculated that the more stable GH levels in the morning hours in men than in women makes a single fasting hs-GH a more accurate measure of GH secretion in men, which, in turn, could explain why associations with various outcomes are stronger in males. Part of the sex discrepancy may also be attributable to power differences, as all outcomes occurred at a higher incidence rate in the male population.

### Study limitations

To date, it is unclear to what extent a fasting value of hs-GH reflects overall GH secretion. The pulsatile mode of secretion is a limitation and makes GH difficult to use in a clinical setting. It is not clear whether our findings represent a linear relationship between GH and the various outcomes, although the quartile analyses were done to evaluate this. Unfortunately, splitting the material caused power difficulties and made a clear-cut answer impossible. An effort to analyze the 10th and 90th percentile versus the middle (data not shown) was also hampered by power, but did not indicate a different association. In addition, we did not measure circulating or tissue insulin-like growth factor-1, which mediates several effects of GH. Despite these issues, we did observe relationships between hs-GH and GH-related phenotypes at baseline as well as associations with long-term outcomes, especially the risk of cardiovascular mortality, suggesting that fasting hs-GH does reflect a medically relevant measure. The association of higher hs-GH values with increased likelihood of a myocardial infarction being fatal may explain why our strongest findings were in the cardiovascular mortality outcome.

Because this was an observational study, we do not prove causation between hs-GH and our various endpoints. Also, the number of nonaccepters to the initial invitation was quite large, which could have biased selection of a more healthy population. Although GH secretion is known to have a strong positive correlation with estradiol levels, we have not excluded pre-menopausal or perimenopausal women from the study (42); this may be a potential confounder in the female analyses. However, the consequence of this would be younger women exhibiting higher GH values, which logically would weaken a positive correlation between hs-GH and the various outcomes. Differences in baseline characteristics in people excluded due to missing plasma samples compared with the study cohort were minor and did not raise any suspicions of selection bias.

## Conclusions

We demonstrate that higher fasting values of hs-GH are associated with cardiovascular morbidity, all-cause mortality, and, in particular, cardiovascular mortality. Further research is needed to elucidate the mechanisms of this association in the general population and its potential implications in the subgroups of patients with a disturbance in GH secretion.

Perspectives **COMPETENCY IN MEDICAL KNOWLEDGE:** In healthy middle-aged people, higher fasting levels of GH are associated with greater cardiovascular morbidity and mortality independent of traditional cardiovascular risk factors.

**COMPETENCY IN PATIENT CARE:** In healthy middle-aged individuals, measurement of fasting GH in plasma may be useful in risk stratification for cardiovascular mortality and aid clinical decision-making for primary prevention of ischemic events.

**TRANSLATIONAL OUTLOOK:** Randomized trials are needed to investigate the effect of GH replacement on cardiovascular outcomes in patients with GHD.
