# Supplementary material for: Fasting Levels of High-Sensitivity Growth Hormone Predict Cardiovascular Morbidity and Mortality: The Malmö Diet and Cancer Study
Source: J Am Coll Cardiol. 2014 Oct 7;64(14):1452–60. doi: 10.1016/j.jacc.2014.03.063 (PMC4180127; doi:10.1016/j.jacc.2014.03.063)
Supplement: Online Data [file mmc1.pdf]

Fasting Levels of High-sensitivity Growth Hormone predicts cardiovascular morbidity and mortality

Supplemental Material

Table of contents

Methods .... p. 2-3

Supplementary tables 1-9 .... p. 4-12

Supplementary figures 1-9 .... p. 12-20

Methods

At the baseline examination blood pressure was measured with a mercury-column sphygmomanometer after 10 minutes rest in the supine position. Waist circumference was measured at the level of the umbilicus. Current cigarette smoking was defined as any use within the last year, and was surveyed through a self-administered questionnaire. The same questionnaire in combination with a diary was used to record prevalence of anti-hypertensive medication at baseline.

CAD was defined on the basis of *International Classification of Diseases,* 9th and 10th revisions (ICD-9 and ICD-10) codes 410 and I21 respectively in the SHDR or SCDR, codes 412 and 414 (ICD-9) or I22, I23 and I25 (ICD-10) of the SCDR. Coronary artery bypass graft surgery (CABG) was identified from national classification of surgical procedures, KKÅ from 1963 until 1989 and Op6 since then. CABG was defined as a procedure code of 3065, 3066, 3068, 3080, 3092, 3105, 3127, 3158 (Op6) or FN (KKÅ97). PCI was defined based on the operation codes FNG05 and FNG02. Heart failure was defined using diagnosis codes 428 (ICD-9) and I50 and I11.0 (ICD-10) as primary diagnosis in the SHDR and SCDR. Stroke was defined on the basis of codes 430, 431, 434 and 436 (ICD-9) or I60, I61, I63 and I64 (ICD-10). Cardiovascular mortality was defined on the basis of (ICD-9) codes 390–459 or (ICD-10) codes I00-I99 in the SCDR.

The following section describes the development of the hs-GH assay. Mouse monoclonal antibodies were raised against GH (NIBSC code 98/574, National Institute for Biological Standards and Control, Herfordshire, UK) (Sphingotec GmbH, Borgsdorf, Germany). The assay was set up as follows: 100 µg of tracer antibody was labeled with 10 µg Acridinium NHS-ester (InVent GmbH, Hennigsdorf, Germany). Labeled antibody was purified by gel-filtration HPLC on Bio-Sil SEC 400-5 (Bio-Rad Laboratories, Inc., USA). The purified

labelled antibody was diluted in (300 mmol/l potassiumphosphate, 100 mmol/l NaCl, 10 mmol/l Na-EDTA, 5 g/l bovine serum albumin, pH 7.0). The final concentration was approx. 600.000 relative light units (RLU) of labelled compound (approx.. 20 ng labeled antibody) per 200 µl. Polystyrene tubes (Greiner Bio-One International AG, Austria) were used as solid phase, on which the solid phase antibody was coated (1.5 µg antibody/0.3 ml 100 mmol/l NaCl, 50 mmol/l Tris/HCl, pH 7.8, for 18 hours). After blocking with 5 % bovine serum albumin, the tubes were washed with PBS, pH 7.4, and vacuum dried. The assay was calibrated using dilutions of GH (NIBSC code 98/574, National Institute for Biological Standards and Control, Herfordshire, UK), diluted in 20 mM K_2PO_4, 6 mM EDTA, 0.5% BSA, 50 µM Amastatin, 100 µM Leupeptin, pH 8.0. The assay was run as follows: 50 µl of sample (or calibrator) and 200 µl labeled antibody were pipetted into antibody coated tubes,and the tubes were incubated for 2 h at 22°C. Unbound tracer was removed by washing 5 times (each 1 ml) with washing solution (20 mmol/l PBS, pH 7.4, 0.1 % Triton X 100). Tube-bound labelled antibody was measured with an AutoLumat LB 953 (Berthold Technologies GmbH & Co. KG).LB 953 . The assay exhibited ideal accuracy (recovery and dilution was within 85% -115% of the expected values).

Table S1. Clinical characteristics of individuals in the database who were excluded from the study (n=1780) compared to the study cohort.

	Excluded	Included
Males, No. (%)	793 (44.8)	1778 (41.1)
Age, mean (SD), years	57.1 (5.8)	57.6 (6.0)
Systolic blood pressure, mean (SD), mmHg	140 (19)	142 (19)
Body Mass Index, Mean (SD), kg/m^2	26.1 (4.1)	25.8 (3.9)
Antihypertensive therapy, No. (%)	347 (19.6)	663 (15.3)
Diabetes Mellitus, No. (%)	127 (10.7)	359 (8.3)
LDL-C†, mean (SD), mmol/L	4.15 (0.99)	4.17 (0.99)
HDL-C†, mean (SD), mmol/L	1.33 (0.38)	1.39 (0.37)
Current smokers, No. (%)	474 (26.8)	1141 (26.4)
Growth Hormone, median (IQR), µg/L, Males	0.10 (0.04-0.33)	0.11 (0.06-0.33)
Growth Hormone, median (IQR), µg/L, Females	0.75 (0.20-3.09)	1.22 (0.40-3.15)

*Total number of individuals available for analysis in the excluded group: Age (n=1771), Gender (n=1771), Systolic Blood pressure (n=1771), BMI (n=1764), Antihypertensive therapy (n=1771), Diabetes Mellitus (n=1186), HDL-C (n=1127), LDL-C (n=1045), Current smoker (n=1771), Growth Hormone (n=187 males, 110 females). Percentage represents the percentage among the individuals available for analysis in each variable.
†Abbreviations: LDL-C, Low-density lipoprotein cholesterol; HDL, High-density lipoprotein cholesterol

Table S2. Results from crude linear regression models examining correlations between fasting values of growth hormone and traditional cardiovascular risk factors.

Risk factor	Male β Coefficient*	Male 95% CI	Male P	Female β Coefficient*	Female 95% CI	Female P
Age	0.02	0.01 to 0.03	<.001	0.00	-0.01 to 0.01	.74
Systolic blood pressure	0.04	-0.01 to 0.09	.09	-0.09	-0.13 to -0.05	<.001
Antihypertensive medication	0.12	-0.01 to 0.25	.07	-0.14	-0.25 to -0.03	.01
BMI†	-0.12	-0.17 to -0.08	<.001	-0.30	-0.34 to -0.27	<.001
Current smoking	0.28	0.18 to 0.38	<.001	0.24	0.15 to 0.32	<.001
LDL-C†	-0.10	-0.15 to -0.06	<.001	-0.12	-0.16 to -0.08	<.001
HDL-C†	0.21	0.17 to 0.26	<.001	0.17	0.13 to 0.21	<.001
Diabetes Mellitus	0.18	0.04 to 0.33	.01	-0.45	-0.61 to -0.30	<.001
Insulin (logarithmic) ‡	-0.14	-0.19 to -0.10	<.001	-0.23	-0.26 to -0.19	<.001
Waist‡	-0.11	-0.16 to -0.06	<.001	-0.32	-0.36 to -0.28	<.001
Body fat percentage‡	-0.10	-0.14 to -0.05	<.001	-0.29	-0.33 to -0.25	<.001
Creatinine‡	-0.03	-0.07 to 0.02	.26	-0.01	-0.05 to 0.03	.51
HbA1c‡	0.10	0.06 to 0.15	<.001	-0.05	-0.09 to -0.01	.007
NT-proBNP (logarithmic) ‡	0.13	0.08 to 0.18	<.001	0.07	0.03 to 0.11	.001

*The β coefficients are expressed as the increment of standardized values of the natural logarithm of hs-GH per 1 increment of standardized values (or prescence of dichotomized risk factor) of the risk factor in question. NB age is not standardized. BMI (weight in kilograms divided by height in meters squared), systolic blood pressure, waist, body fat percentage and fasting values of HDL-C, LDL-C, creatinine and natural logarithm of insulin and NT-proBNP are standardized. Prevalence of Diabetes mellitus, current smoking and use of antihypertensive medication are dichotomous variables.

†Abbreviations: BMI, Body Mass Index; LDL-C, Low-density lipoprotein cholesterol; HDL-C, High-density lipoprotein cholesterol, NT-proBNP, N-terminal prohormone of brain natriuretic peptide.

‡Due to missing values, fewer persons available for analysis in: Insulin (2498 females, 1745 males), waist (2545 females, 1777 males), body fat percentage (2545 females, 1774 males), Creatinine (2500 females, 1750 males), HbA1c (2541 females, 1768 males), NT-proBNP (2399 females, 1647 males).

Table S3. Results from simple and multiple linear regression models examining correlations between fasting values of growth hormone and traditional cardiovascular risk factors. Not gender separated.

	Crude			Adjusted		
Risk factor	β Coefficient*	95% CI	P	β Coefficient*	95% CI	P
Gender	NA	NA	NA	-0.12	-0.18 to -0.05	<.001
Age	0.01	0.00 to 0.01	<.001	0.02	0.01 to 0.02	<.001
Systolic blood pressure	-0.04	-0.07 to -0.01	.01	-0.01	-0.05 to 0.02	.40
Antihypertensive medication	-0.03	-0.12 to 0.05	.43	0.10	0.02 to 0.18	.02
BMI†	-0.24	-0.27 to -0.21	<.001	-0.19	-0.22 to -0.16	<.001
Current smoking	0.25	0.19 to 0.32	<.001	0.23	0.17 to 0.30	<.001
LDL-C†	-0.11	-0.14 to -0.09	<.001	-0.10	-0.13 to -0.07	<.001
HDL-C†	0.17	0.14 to 0.20	<.001	0.14	0.10 to 0.17	<.001
Diabetes Mellitus	-0.11	-0.22 to -0.00	.04	0.06	-0.05 to 0.17	.28

*The β coefficients are expressed as the increment of standardized values of the natural logarithm of hs-GH per 1 increment of standardized values (or prescence of dichotomized risk factor) of the risk factor in question. NB age is not standardized. BMI (weight in kilograms divided by height in meters squared), systolic blood pressure and fasting values of HDL-C and LDL-C are standardized. Prevalence of Diabetes mellitus, current smoking and use of antihypertensive medication are dichotomous variables.

Values of hs-GH are standardized separately in men and women and this same gender-specific standardization is used in the combined analyses.

†Abbreviations: BMI, Body Mass Index; LDL-C, Low-density lipoprotein cholesterol; HDL-C, High-density lipoprotein cholesterol

Table S4. Results from multiple linear regression models examining correlations between fasting values of growth hormone and traditional cardiovascular risk factors. Addition of other risk factors.

	Male			Female		
Risk factor†	β Coefficient*	95% CI	P	β Coefficient*	95% CI	P
Age	0.02	0.01 to 0.03	<.001	0.02	0.01 to 0.02	<.001
Systolic blood pressure	0.02	-0.03 to 0.07	.38	-0.04	-0.08 to 0.00	.04
Antihypertensive medication	0.13	0.00 to 0.26	.05	0.07	-0.03 to 0.18	.18
BMI‡	-0.07	-0.12 to -0.03	.003	-0.25	-0.29 to -0.21	<.001
Current smoking	0.29	0.19 to 0.39	<.001	0.19	0.10 to 0.27	<.001
LDL-C‡	-0.09	-0.13 to -0.04	<.001	-0.10	-0.14 to -0.06	<.001
HDL-C‡	0.20	0.15 to 0.25	<.001	0.08	0.04 to 0.12	<.001
Diabetes Mellitus	0.21	0.07 to 0.36	.004	-0.16	-0.31 to 0.00	.05
Bodyfat percentage	-0.04	-0.10 to 0.01	.15	-0.11	-0.18 to -0.04	.003
Waist	-0.04	-0.14 to 0.05	.38	-0.19	-0.27 to -0.12	<.001
NT-proBNP‡	0.08	0.03 to 0.13	<.001	0.03	-0.01 to 0.07	.11
Creatinine	-0.01	-0.06 to 0.03	.55	-0.01	-0.05 to 0.03	.57
HbA1c	0.09	0.04 to 0.14	.001	0.02	-0.03 to 0.06	.41
Insulin	-0.10	-0.16 to -0.05	<.001	-0.09	-0.13 to -0.04	<.001

*The β coefficients are expressed as the increment of standardized values of the natural logarithm of hs-GH per 1 increment of standardized values (or prescence of dichotomized risk factor) of the risk factor in question. NB age is not standardized. BMI (weight in kilograms divided by height in meters squared), systolic blood pressure, and fasting values of HDL-C, LDL-C, creatinine, HbA1c, waist, bodyfat percentage and natural logarithm of insulin and NT-proBNP are standardized. Prevalence of Diabetes mellitus, current smoking and use of antihypertensive medication are dichotomous variables.

†The values for the first eight risk factors are from a linear regression model including only these variables. The other six risk factors, which are not included in the basic model, were then one at a time added to the basic model. In these extra models, only the values for the newly added variable are shown.

‡Abbreviations: BMI, Body Mass Index; LDL-C, Low-density lipoprotein cholesterol; HDL-C, High-density lipoprotein cholesterol, NT-proBNP, N-terminal prohormone of brain natriuretic peptide.

Due to missing values, fewer persons available for analysis in: Insulin (2498 females, 1745 males), waist (2545 females, 1777 males), body fat percentage (2545 females, 1774 males), Creatinine (2500 females, 1750 males), HbA1c (2541 females, 1768 males), NT-proBNP (2399 females, 1647 males).

Table S5: Multivariate adjusted Cox proportional hazards models for baseline fasting value of hs-GH vs incidence of CAD, stroke, congestive heart failure, all-cause mortality and cardiovascular mortality.

			Basic model			+Insulin			+NT-proBNP			+HbA1c		
**Event**	**Subgroup**	**events[‡]**	**HR***	**95% CI**	**P**	**HR***	**95% CI**	**P**	**HR***	**95% CI**	**P**	**HR***	**95% CI**	**P**
**CAD[†]**	All	397	1.11	1.01-1.23	.04	1.12	1.01-1.24	.03	1.10	0.99-1.22	.08	1.08	0.98-1.20	.12
	Males	247	1.17	1.04-1.33	.01	1.17	1.03-1.32	.01	1.14	1.00-1.30	.05	1.15	1.01-1.30	.03
	Females	150	1.02	0.86-1.21	.81	1.03	0.86-1.23	.73	1.05	0.88-1.25	.59	1.00	0.84-1.19	.96
**Stroke**	All	251	1.18	1.04-1.34	.01	1.18	1.03-1.34	.01	1.16	1.01-1.32	.03	1.18	1.04-1.34	.01
	Males	132	1.21	1.02-1.43	.02	1.21	1.02-1.43	.03	1.22	1.02-1.45	.03	1.21	1.03-1.43	.02
	Females	119	1.16	0.95-1.41	.15	1.15	0.94-1.40	.18	1.11	0.91-1.36	.32	1.15	0.94-1.41	.30
**Congestive**	All	107	1.25	1.03-1.52	.02	1.22	1.01-1.49	.04	1.23	1.00-1.50	.05	1.24	1.02-1.51	.03
**Heart**	Males	156	1.07	0.83-1.39	.59	1.04	0.79-1.35	.80	1.05	0.80-1.39	.72	1.05	0.80-1.36	.74
**Failure**	Females	51	1.48	1.08-2.04	.02	1.47	1.07-2.02	.02	1.43	1.04-1.98	.03	1.48	1.08-2.04	.02
**Total**	All	645	1.17	1.08-1.26	<.001	1.16	1.07-1.26	<.001	1.17	1.08-1.27	<.001	1.15	1.06-1.25	<.001
**mortality**	Males	341	1.25	1.13-1.39	<.001	1.24	1.12-1.37	<.001	1.26	1.13-1.40	<.001	1.23	1.11-1.37	<.001
	Females	304	1.04	0.92-1.18	.50	1.05	0.93-1.19	.46	1.06	0.93-1.20	.38	1.04	0.92-1.17	.56
**Cardiovascular**	All	186	1.43	1.24-1.66	<.001	1.42	1.22-1.64	<.001	1.45	1.24-1.69	<.001	1.40	1.21-1.62	<.001
**mortality**	Males	105	1.44	1.20-1.72	<.001	1.41	1.17-1.69	<.001	1.46	1.21-1.77	<.001	1.40	1.17-1.68	<.001
	Females	81	1.45	1.12-1.88	.004	1.47	1.13-1.91	.004	1.46	1.12-1.92	.006	1.44	1.11-1.87	.006

*Hazard ratios (HR) (95% CI) are expressed per 1 SD increment of the natural logarithm of hs-GH. Basic models adjusted for: age, systolic blood pressure, use of antihypertensive medication, BMI (weight in kilograms divided by height in meters squared), Prevalence of diabetes mellitus, current smoking and fasting values of HDL-C and LDL-C. Adjusted for sex in analyses including both genders. In the 3 additional models Insulin, NT-proBNP or HbA1c are added to the basic variables. Natural logarithms of Insulin and NT-proBNP are used.

Values of hs-GH are standardized separately in men and women and this same gender-specific standardization is used in the combined analyses.

[†]Abbreviations: CAD, coronary artery disease; NT-proBNP, N-terminal prohormone of brain natriuretic peptide.

[‡]Number of events for basic models, may be fewer in the additional models since fewer individuals are available for analysis. 4323 individuals in basic model (1778 males, 2545 females). Fewer available in the other analyses due to missing values: 80 individuals (33 males, 47 females) missing in Insulin, 277 individuals (131 males, 146 females) missing in NT-proBNP, 14 individuals (10 males, 4 females) missing in HbA1c.

Table S6: Multivariate adjusted Cox proportional hazards models for baseline fasting value of hs-GH vs incidence of CAD, stroke, congestive heart failure, all-cause mortality and cardiovascular mortality.

			Basic model			+Creatinine			+Waist			+Bodyfat percentage		
**Event**	**Subgroup**	**events[‡]**	**HR***	**95% CI**	**P**	**HR***	**95% CI**	**P**	**HR***	**95% CI**	**P**	**HR***	**95% CI**	**P**
**CAD[†]**	All	397	1.11	1.01-1.23	.04	1.11	1.00-1.23	.05	1.11	1.01-1.23	.03	1.11	1.01-1.23	.04
	Males	247	1.17	1.04-1.33	.01	1.17	1.04-1.33	.01	1.17	1.04-1.33	.01	1.17	1.04-1.33	.01
	Females	150	1.02	0.86-1.21	.81	1.01	0.85-1.20	.94	1.04	0.87-1.23	.68	1.02	0.86-1.21	.81
**Stroke**	All	251	1.18	1.04-1.34	.01	1.17	1.03-1.33	.02	1.18	1.04-1.34	.01	1.18	1.04-1.34	.01
	Males	132	1.21	1.02-1.43	.02	1.19	1.01-1.41	.04	1.22	1.03-1.44	.02	1.21	1.03-1.43	.02
	Females	119	1.16	0.95-1.41	.15	1.17	0.96-1.42	.13	1.17	0.96-1.42	.13	1.16	0.96-1.42	.13
**Congestive**	All	107	1.25	1.03-1.52	.02	1.23	1.01-1.50	.04	1.26	1.04-1.52	.02	1.25	1.03-1.52	.02
**Heart**	Males	156	1.07	0.83-1.39	.59	1.05	0.80-1.37	.74	1.06	0.82-1.37	.67	1.07	0.83-1.39	.59
**Failure**	Females	51	1.48	1.08-2.04	.02	1.46	1.06-2.01	.02	1.54	1.11-2.13	.009	1.50	1.09-2.07	.01
**Total mortality**	All	645	1.17	1.08-1.26	<.001	1.15	1.06-1.25	<.001	1.17	1.08-1.27	<.001	1.17	1.08-1.26	<.001
	Males	341	1.25	1.13-1.39	<.001	1.24	1.11-1.37	<.001	1.25	1.13-1.38	<.001	1.25	1.13-1.38	<.001
	Females	304	1.04	0.92-1.18	.50	1.04	0.92-1.17	.58	1.06	0.93-1.20	.37	1.05	0.93-1.18	.46
**Cardiovascular**	All	186	1.43	1.24-1.66	<.001	1.42	1.22-1.64	<.001	1.43	1.24-1.66	<.001	1.43	1.24-1.65	<.001
**mortality**	Males	105	1.44	1.20-1.72	<.001	1.41	1.17-1.69	<.001	1.43	1.20-1.71	<.001	1.43	1.20-1.71	<.001
	Females	81	1.45	1.12-1.88	.004	1.46	1.13-1.90	.004	1.46	1.13-1.89	.004	1.46	1.13-1.89	.004

*Hazard ratios (HR) (95% CI) are expressed per 1 SD increment of the natural logarithm of hs-GH. Basic models adjusted for: age, systolic blood pressure, use of antihypertensive medication, BMI (weight in kilograms divided by height in meters squared), Prevalence of diabetes mellitus, current smoking and fasting values of HDL-C and LDL-C. Adjusted for sex in analyses including both genders. In the 3 additional models creatinine, waist or bodyfat percentage are added to the basic variables.

Values of hs-GH are standardized separately in men and women and this same gender-specific standardization is used in the combined analyses.

[†]Abbreviations: CAD, coronary artery disease

Number of events for basic models, may be fewer in the additional models since fewer individuals are available for analysis. 4323 individuals in basic model (1778 males, 2545 females). Fewer available in the other analyses due to missing values: 73 individuals (28 males, 45 females) in Creatinine, 1 male missing in waist, 4 males missing in bodyfat percentage.

Table S7: Crude cox proportional hazards models for baseline fasting value of hs-GH vs incidence of CAD, stroke, all-cause mortality and cardiovascular mortality. No other variable included in the model

Event	Subgroup	n/events	HR*	95% CI	P
**CAD**[†]	All	4323/397	1.07	0.97-1.19	.15
	Males	1778/247	1.17	1.04-1.31	.01
	Females	2545/150	0.95	0.81-1.11	.50
**Stroke**	All	4323/251	1.16	1.03-1.31	.02
	Males	1778/132	1.26	1.08-1.48	.003
	Females	2545/119	1.04	0.87-1.25	.64
**Congestive Heart Failure**	All	4323/107	1.25	1.03-1.50	.02
	Males	1778/56	1.26	0.99-1.60	.06
	Females	2545/51	1.23	0.92-1.63	.15
**Total mortality**	All	4323/645	1.21	1.12-1.31	<.001
	Males	1778/341	1.36	1.24-1.49	<.001
	Females	2545/304	1.04	0.93-1.16	.53
**Cardiovascular mortality**	All	4323/186	1.48	1.28-1.70	<.001
	Males	1778/105	1.54	1.31-1.82	<.001
	Females	2545/81	1.32	1.05-1.65	.02

*Hazard ratios (HR) (95% CI) are expressed per 1 SD increment of the natural logarithm of hs-GH. Values of hs-GH are standardized separately in men and women and this same gender-specific standardization is used in the combined analyses.

[†]Abbreviations: CAD, coronary artery disease

Table S8: Improvement of discrimination (C-statistics) and reclassification of 10-year risk estimates (category-free Net Reclassification Improvement (NRI (>0))) for addition of growth hormone to the Framingham model of 10-year hard coronary heart disease.

		NRI (>0)†			C-statistics‡	
		**Overall NRI (95%CI)**	**Events (95%CI)**	**Nonevents (95%CI)**	**Framingham(95%CI)**	**+GH (95%CI)**
**CAD**	All	0.018 (-0.061 to 0.140)	-	-	0.690 (0.666-0.715)	0.691 (0.666-0.716)
	Male	0.118 (-0.020 to 0.292)	-	-	0.642 (0.610-0.676)	0.646 (0.612-0.679)
	Female	-0.070 (-0.167 to 0.210)	-	-	0.708 (0.668-0.747)	0.707 (0.668-0.747)
**Stroke**	All	0.151 (0.021 to 0.308)	0.054 (-0.030 to 0.180)	0.096 (0.047 to 0.149)	0.723 (0.695-0.751)	0.726 (0.698-0.754)
	Male	0.195 (0.031 to 0.351)	-0.077 (-0.182 to 0.060)	0.272 (0.190 to 0.339)	0.722 (0.684-0.759)	0.727 (0.689-0.764)
	Female	0.122 (-0.120 to 0.344)	-	-	0.706 (0.664-0.747)	0.707 (0.666-0.748)
**CHF**	All	0.163 (-0.025 to 0.325)	-	-	0.722 (0.677-0.767)	0.731 (0.686-0.776)
	Male	0.128 (-0.065 to 0.421)	-	-	0.673 (0.610-0.736)	0.681 (0.619-0.744)
	Female	0.129 (-0.238 to 0.467)	-	-	0.750 (0.682-0.817)	0.762 (0.694-0.830)
**Total**	All	0.220 (0.021 to 0.434)	0.086 (-0.066 to 0.259)	0.135 (0.087 to 0.203)	0.689 (0.669-0.709)	0.696 (0.676-0.717)
**Mortality**	Male	0.542 (0.152 to 1.001)	0.120 (-0.149 to 0.486)	0.422 (0.288 to 0.606)	0.678 (0.651-0.706)	0.695 (0.668-0.722)
	Female	0.045 (-0.044 to 0.202)	-	-	0.681 (0.651-0.711)	0.682 (0.652-0.712)
**Cardiovascular**	All	0.574 (0.227 to 0.912)	0.397 (0.063 to 0.690)	0.177 (0.067 to 0.286)	0.762 (0.730-0.793)	0.774 (0.742-0.806)
**Mortality**	Male	0.903 (0.220 to 1.932)	0.472 (-0.092 to 1.489	0.431 (0.292 to 0.634)	0.732 (0.686-0.779)	0.747 (0.701-0.794)
	Female	0.422 (0.179 to 0.622)	0.356 (0.174 to 0.477)	0.067 (-0.023 to 0.185)	0.773 (0.727-0.819)	0.784 (0.736-0.833)

*Variables included in the original model: Age, systolic blood pressure, total cholesterol, Antihypertensive medication, current smoking and high-density lipoprotein cholesterol (HDL-C).

† Data in "events" and "nonevents" are only shown in the outcomes where the basic overall NRI is significant.

‡ Harrell's C. Area under the ROC (Receiver operating characteristics) curve in basic Framingham model without hs-GH (basic) and the Framingham basic model with addition of hs-GH (+GH).

Abbreviations: CAD, coronary artery disease; CHF, congestive heart failure; NRI, Net Reclassification Improvement.

Table S9: Clinical characteristics of the study population divided into gender-specific quartiles based on fasting growth hormone values.

	Q1*	Q2*	Q3*	Q4*
Age, mean (SD), years	57.0 (5.9)	57.8 (6.0)	57.9 (6.0)	57.8 (6.0)
Systolic blood pressure, mean (SD), mmHg	143 (19)	142 (19)	141 (20)	141 (19)
Body Mass Index, Mean (SD), kg/m^2	27.1 (4.0)	26.0 (3.8)	25.3 (3.7)	24.7 (3.8)
Antihypertensive therapy, %	17.0	14.2	15.3	15.1
Diabetes Mellitus, %	10.7	7.6	7.3	7.9
LDL-C†, mean (SD), mmol/L	4.30 (0.97)	4.25 (1.00)	4.11 (1.01)	4.01 (0.95)
HDL-C†, mean (SD), mmol/L	1.31 (0.34)	1.37 (0.36)	1.42 (0.37)	1.46 (0.38)
Current smokers, %	21.3	22.7	28.0	33.5
Growth Hormone, µg/L, range, males	0.02-0.05	0.06-0.11	0.12-0.33	0.34-23.94
Growth Hormone, µg/L, range, females	0.01-0.39	0.40-1.21	1.22-3.14	3.15-40.60

*Quartile 1 (Q1) represents the quartile with the lowest values of fasting hs-GH. Males and females are divided into the quartiles separately, which makes the male/female ratio similar in the quartiles, but the cut-off values different in the different genders.

†Abbreviations: LDL-C, Low-density lipoprotein cholesterol; HDL, High-density lipoprotein cholesterol

Supplemental figure 1: Kaplan-Meier plot showing one minus cumulative CAD event-free survival in the whole cohort during 17 years of follow-up in quartiles of the baseline fasting plasma concentration of hs-GH. Concentrations of Q1-Q4 are given in Supplementary table 2

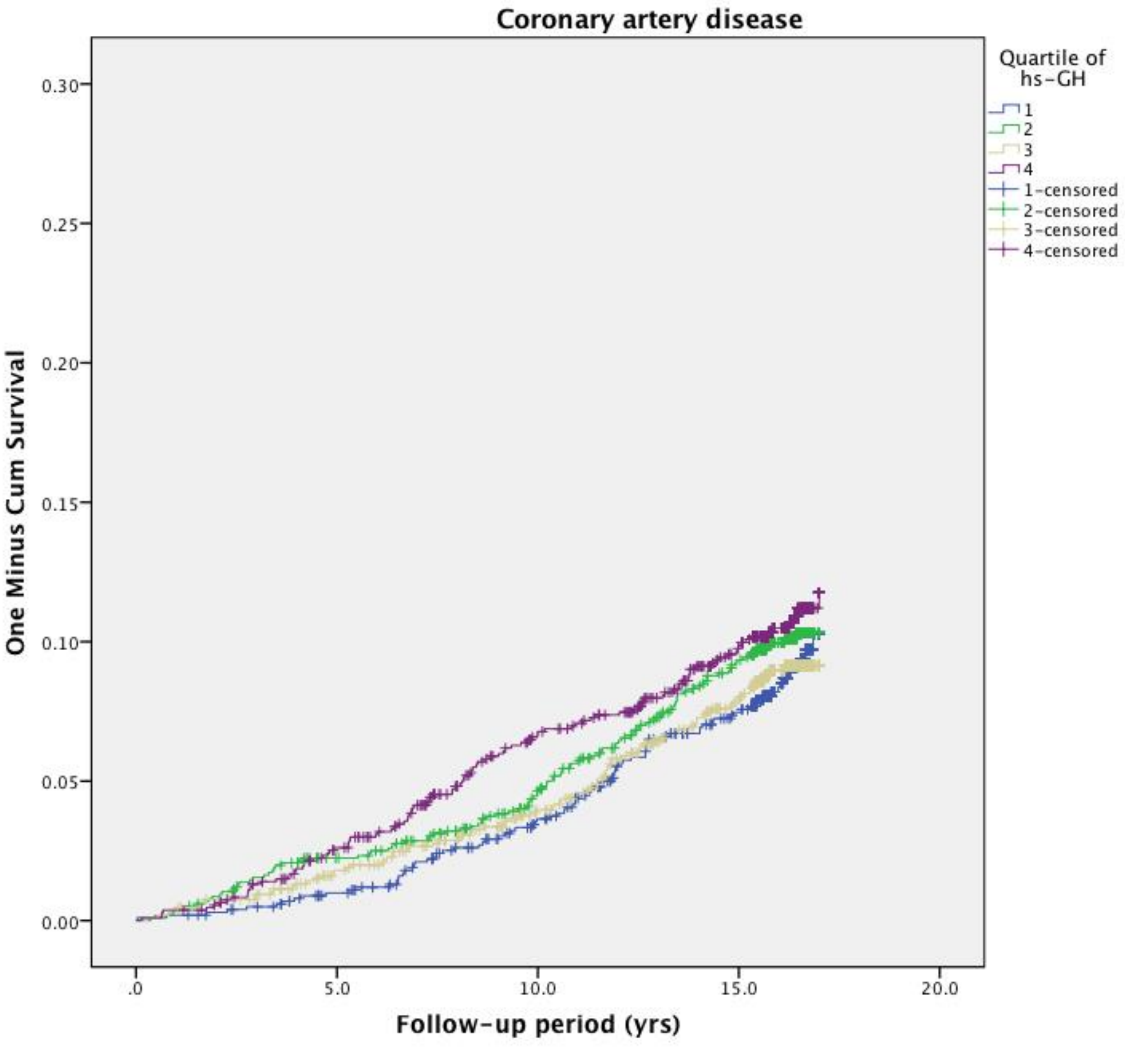

Quartile 1 (Q1) represents the quartile with the lowest values of fasting hs-GH. Males and females are divided into the quartiles separately, which makes the male/female ratio similar in the quartiles, but the cut-off values different in the different genders.

Supplemental figure 2: Kaplan-Meier plot showing one minus cumulative CAD event-free survival for males during 17 years of follow-up in quartiles of the baseline fasting plasma concentration of hs-GH. Concentrations of Q1-Q4 are given in Supplementary table 2.

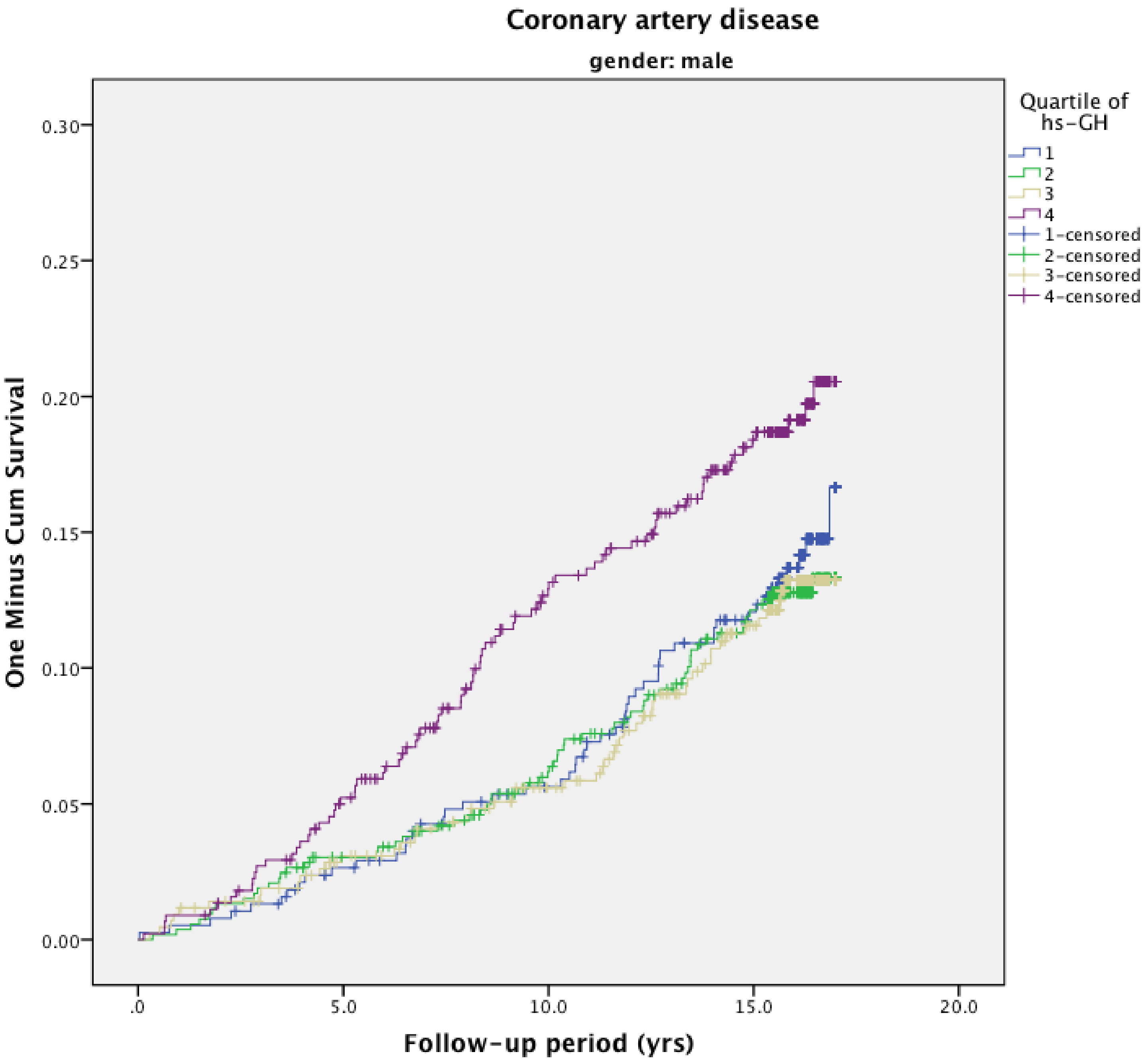

Quartile 1 (Q1) represents the quartile with the lowest values of fasting hs-GH.

Supplemental figure 3: Kaplan-Meier plot showing one minus cumulative CAD event-free survival for females during 17 years of follow-up in quartiles of the baseline fasting plasma concentration of hs-GH. Concentrations of Q1-Q4 are given in Supplementary table 2.

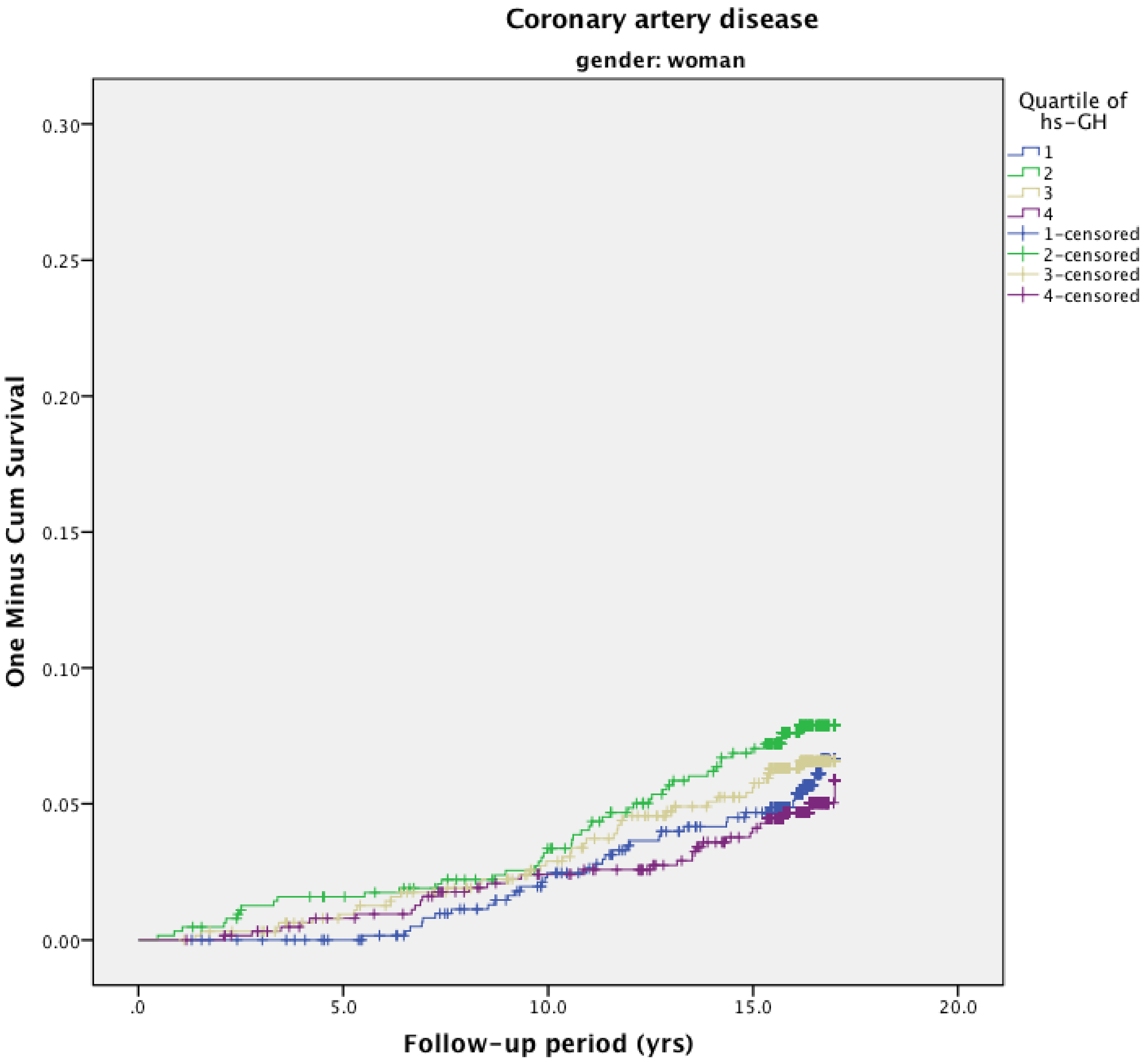

†Quartile 1 (Q1) represents the quartile with the lowest values of fasting hs-GH.

Supplemental figure 4: Kaplan-Meier plot showing one minus cumulative survival in the whole cohort during 17 years of follow-up in quartiles of the baseline fasting plasma concentration of hs-GH. Concentrations of Q1-Q4 are given in Supplementary table 2.

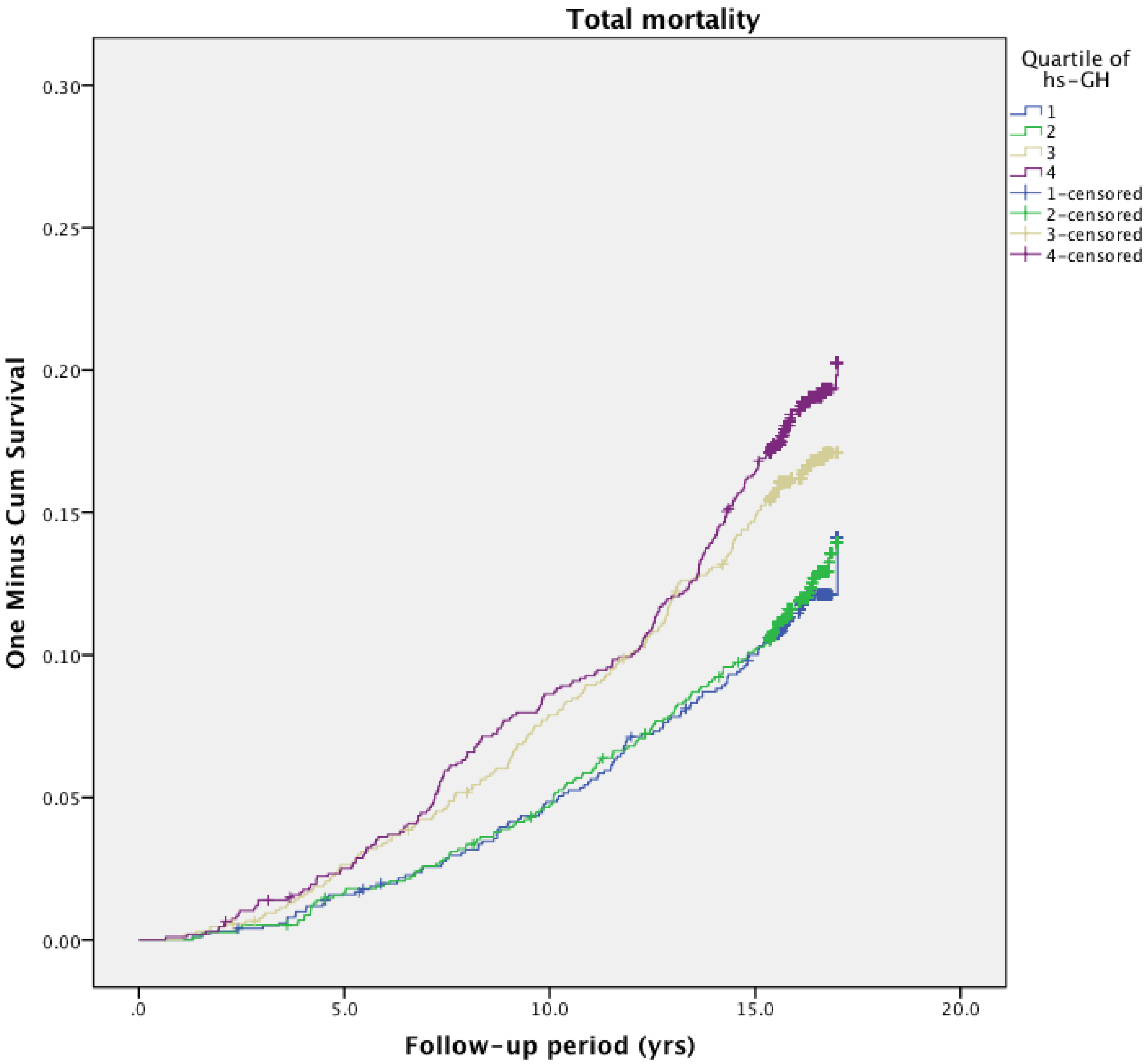

Quartile 1 (Q1) represents the quartile with the lowest values of fasting hs-GH. Males and females are divided into the quartiles separately, which makes the male/female ratio similar in the quartiles, but the cut-off values different in the different genders.

Supplemental figure 5: Kaplan-Meier plot showing one minus cumulative survival for males during 17 years of follow-up in quartiles of the baseline fasting plasma concentration of hs-GH. Concentrations of Q1-Q4 are given in Supplementary table 2.

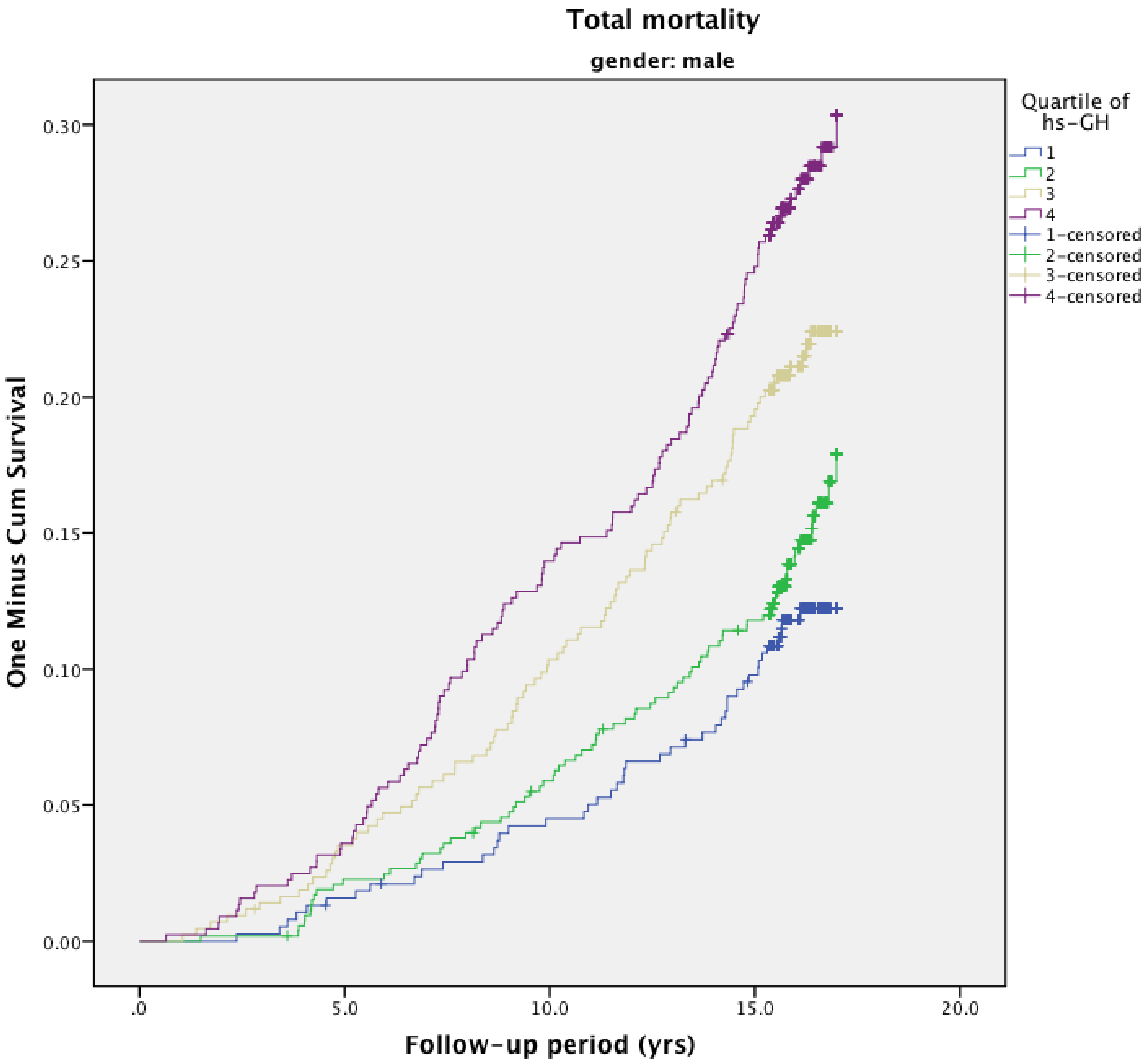

Quartile 1 (Q1) represents the quartile with the lowest values of fasting hs-GH.

Supplemental figure 6: Kaplan-Meier plot showing one minus cumulative survival for females during 17 years of follow-up in quartiles of the baseline fasting plasma concentration of hs-GH. Concentrations of Q1-Q4 are given in Supplementary table 2.

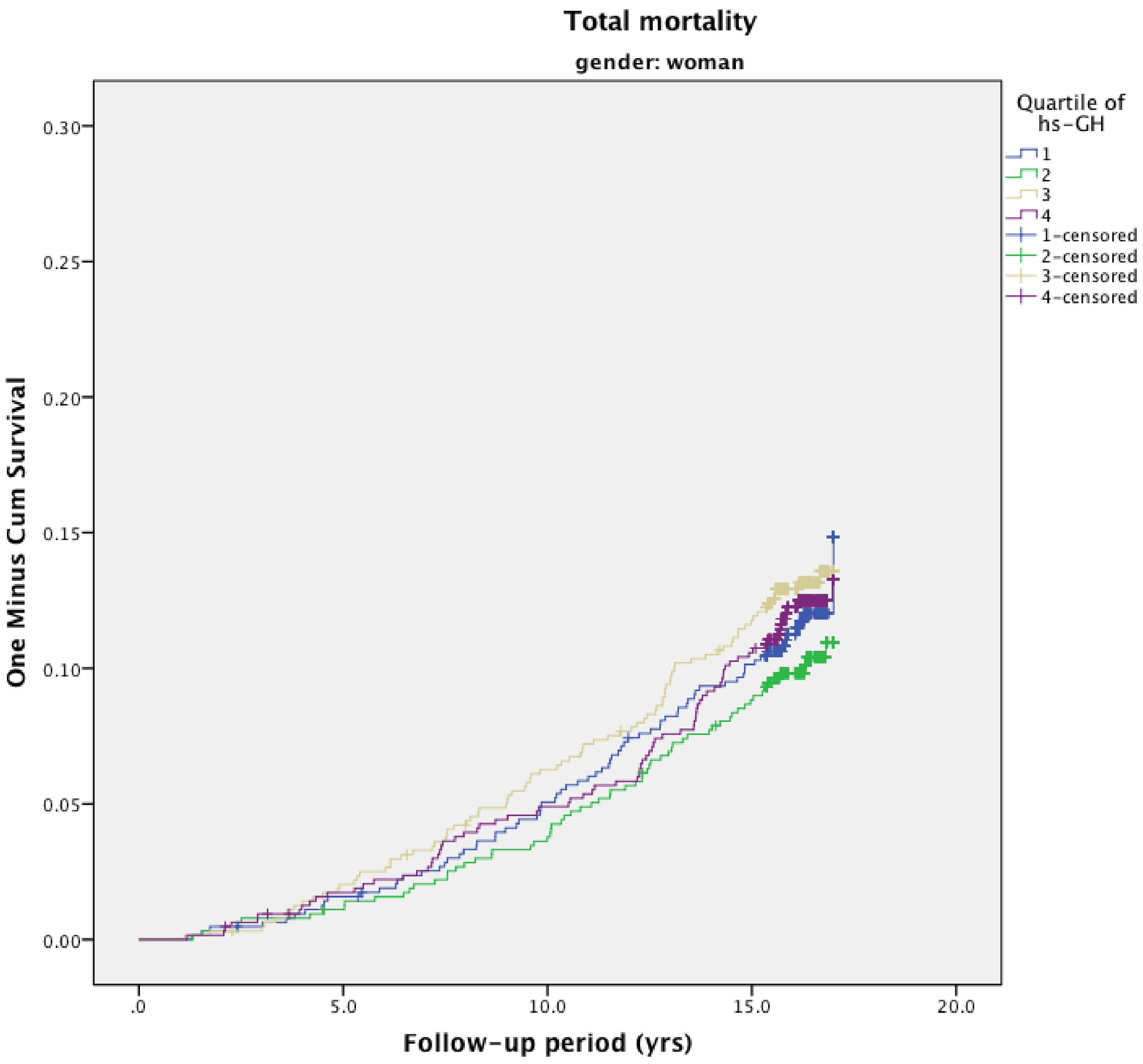

Quartile 1 (Q1) represents the quartile with the lowest values of fasting hs-GH.

Supplemental figure 7: Kaplan-Meier plot showing cumulative cardiovascular mortality in the whole cohort during 17 years of follow-up in quartiles of the baseline fasting plasma concentration of hs-GH. Concentrations of Q1-Q4 are given in Supplementary table 2.

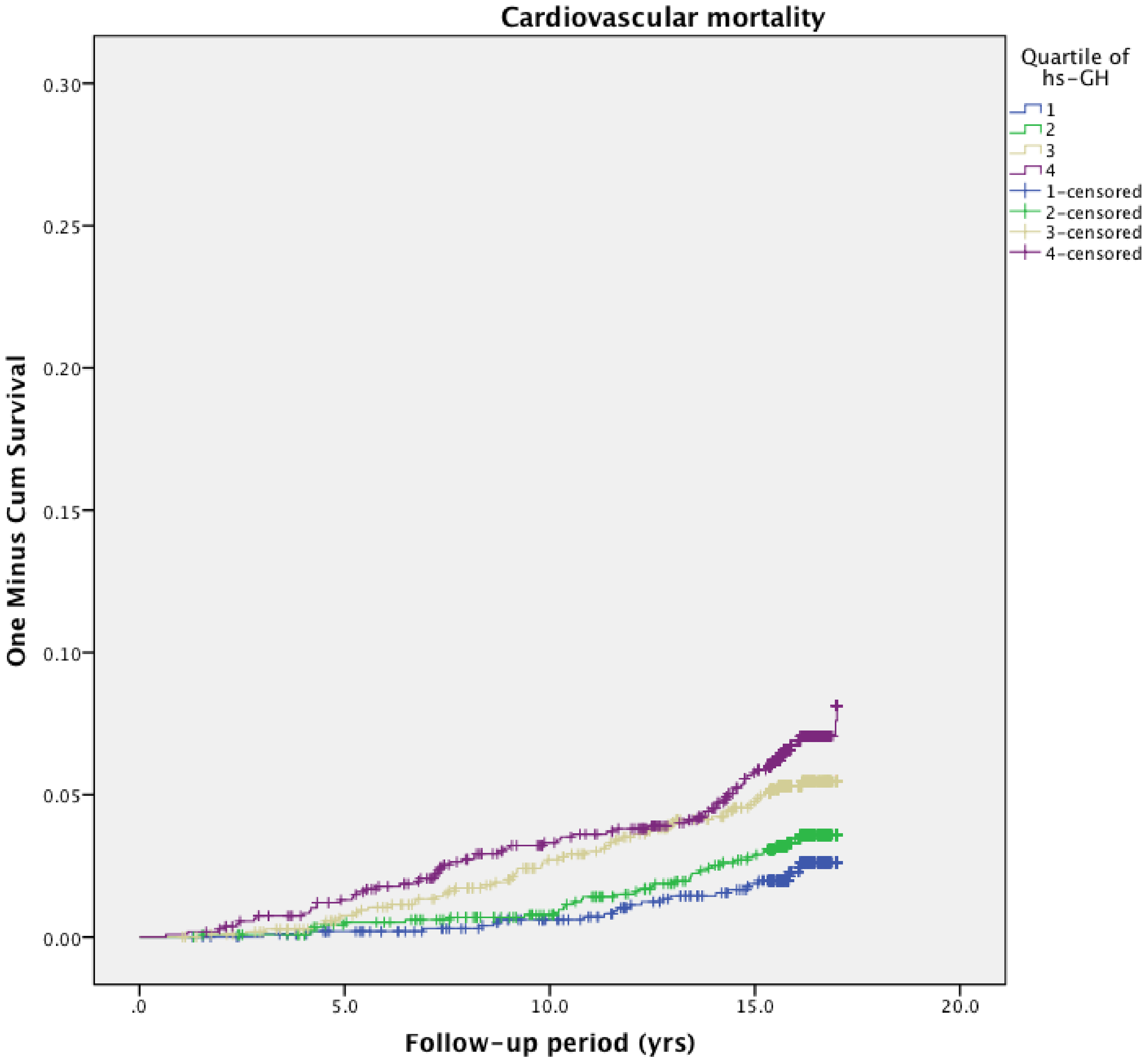

Quartile 1 (Q1) represents the quartile with the lowest values of fasting hs-GH. Males and females are divided into the quartiles separately, which makes the male/female ratio similar in the quartiles, but the cut-off values different in the different genders.

Supplemental figure 8: Kaplan-Meier plot showing cumulative cardiovascular mortality for males during 17 years of follow-up in quartiles of the baseline fasting plasma concentration of hs-GH. Concentrations of Q1-Q4 are given in Supplementary table 2.

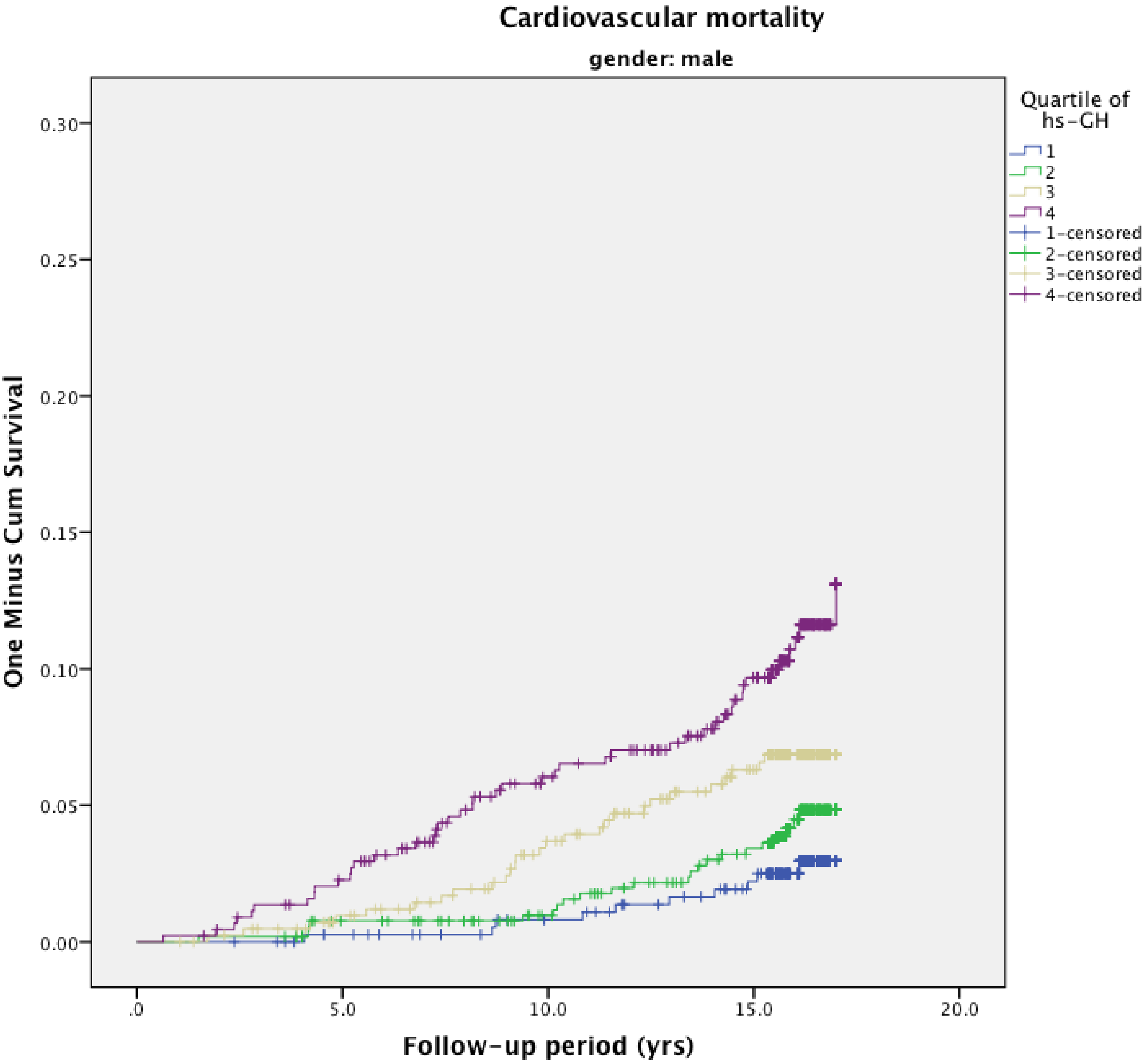

Quartile 1 (Q1) represents the quartile with the lowest values of fasting hs-GH.

Supplemental figure 9: Kaplan-Meier plot showing cumulative cardiovascular mortality for females during 17 years of follow-up in quartiles of the baseline fasting plasma concentration of hs-GH. Concentrations of Q1-Q4 are given in Supplementary table 2.

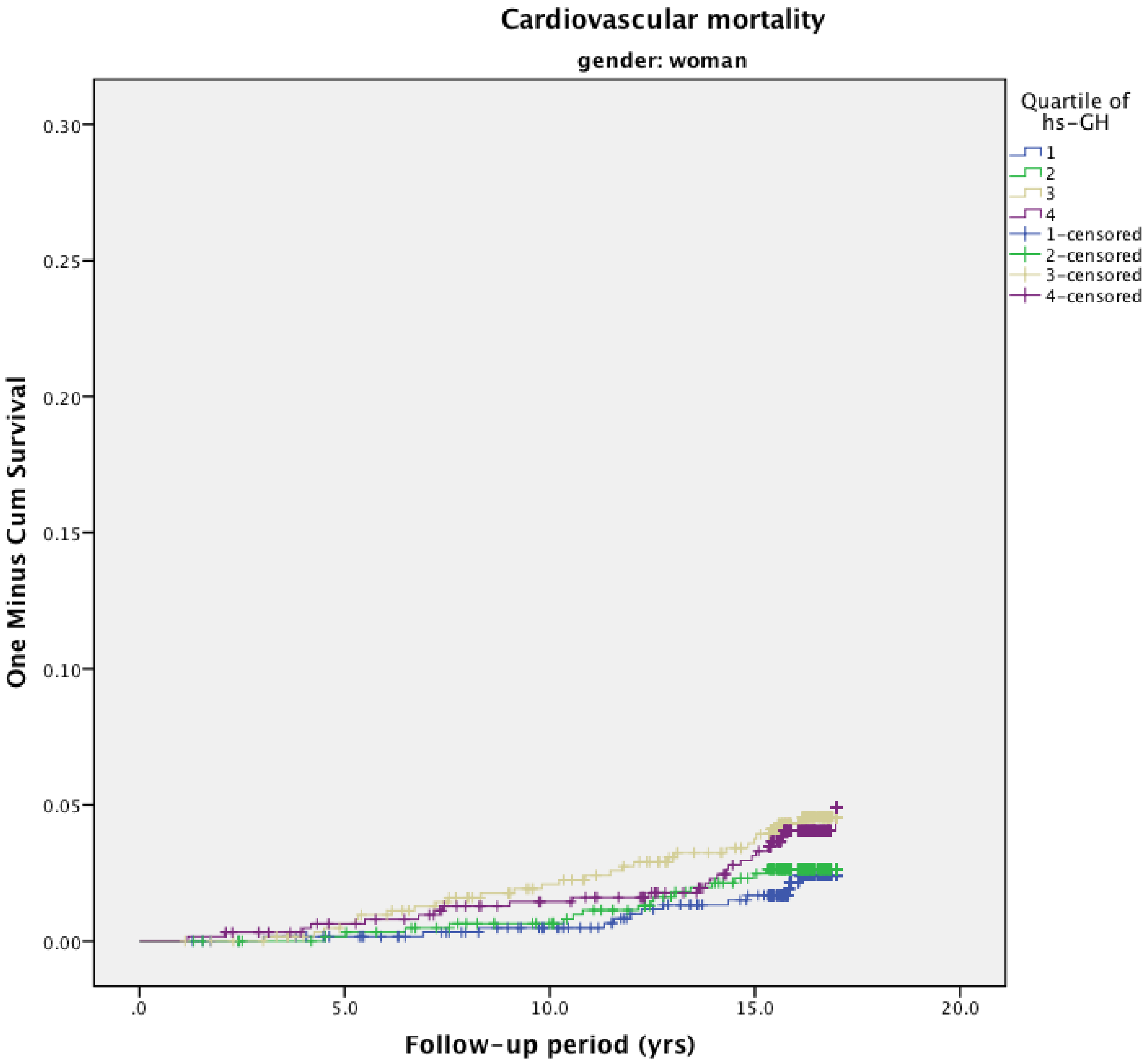

Quartile 1 (Q1) represents the quartile with the lowest values of fasting hs-GH.
